# Synthèse et étude structrale de lyonsite-type (Na_0,4_,Li_0,6_)(Fe,Li_2_)(MoO_4_)_3_

**DOI:** 10.1107/S2056989015008737

**Published:** 2015-05-09

**Authors:** Amira Souilem, Mohamed Faouzi Zid, Ahmed Driss

**Affiliations:** a Laboratoire de Matériaux et Cristallochimie, Faculté des Sciences de Tunis, Université de Tunis ElManar, 2092 Manar II Tunis, Tunisie

**Keywords:** crystal structure, lyonsite-type, monovalent cation molybdate, bond-valence calculations

## Abstract

The new compound (Na_0.4_,Li_0.6_)(Fe,Li_2_)(MoO_4_)_3_ was synthesized by cooling from the melt. Its anionic framework is built up from two distinct *M*O_6_ octa­hedra, each containing disordered Li^+^ and Fe^3+^ ions in 0.6:0.4 and 0.7:0.3 ratios, and two MoO_4_ tetra­hedra, which link by vertex-sharing of their O atoms. These tetra­meric units are further linked by sharing edges between octa­hedra and by formation of *M*—O—Mo (*M* = Fe/Li) bridges, forming ribbons propagating in the [100] direction. The ribbons are cross-linked in both the *b*- and *c*-axis directions, giving rise to a three-dimensional framework having [100] tunnels in which the monovalent Na^+^/Li^+^ cations (0.4:0.6 ratio) lie. Bond-valence calculations are consistent with the disorder model for the cations. The structure of the title compound, which is isotypic with Li_3_Fe(MoO_4_)_3_ and Li_3_Ga(MoO_4_)_3_, is compared briefly with those of LiFeMo_2_O_8_ and Li_1.6_Mn_2.2_(MoO_4_)_3_.

## Abstract 2

The anionic framework of (Na_0.4_,Li_0.6_)(Fe,Li_2_)(MoO_4_)_3_ is built up from two distinct MO_6_ octa­hedra, each containing disordered Li^+^ and Fe^3+^ ions, and two MoO_4_ tetra­hedra, which link by vertex-sharing of their O atoms. These tetra­meric units are further linked by sharing edges between octa­hedra and by formation of *M*—O—Mo (*M* = Fe/Li) bridges, forming ribbons propagating in the [100]. The ribbons are cross-linked in both the *b*- and *c*-axis directions, giving rise to a three-dimensional framework having [100] tunnels in which the monovalent Na^+^/Li^+^ cations lie.

## Contexte chimique   

La famille des molybdates de cations monovalents est l’objet d’un grand intérêt ces dernières années à cause de l’importance de leurs propriétés physiques et les applications potentielles, prenant l’exemple des matériaux laser prometteurs, des luminophores efficaces, qui sont caractérisés par une longue durée de vie et une haute intensité de luminescence, ainsi que des matériaux ferroélectriques et anti­ferro­magnétiques (Morozov *et al.*, 2003 ▸; Isupov, 2005 ▸; Maczka *et al.*, 2005 ▸; Jorge *et al.*, 2004 ▸). L’étude des structures cristallines de ces molybdates souligne leur polymorphisme dépendant de la température (Klevtsov & Klevtsova, 1977 ▸). De plus, les composés à base de polyanions sont actuellement proposés comme une alternative prometteuse aux matériaux de Li*M*O_2_ (*M* = Ni, Co, Mn) stratifiés comme cathode pour des piles rechargeables. L’utilisation du lithium est visé vu qu’il possède le potentiel électrochimique le plus élevé par rapport à l’électrode à hydrogène standard ce qui confère à la batterie une plus haute tension d’où la naissance des batteries rechargeables au lithium (LiFePO_4_) (Padhi *et al.*, 1997 ▸) qui sont un dispositif important pour le stockage de l’énergie électrique.

## Commentaire structurelle   

**Figure 1:**
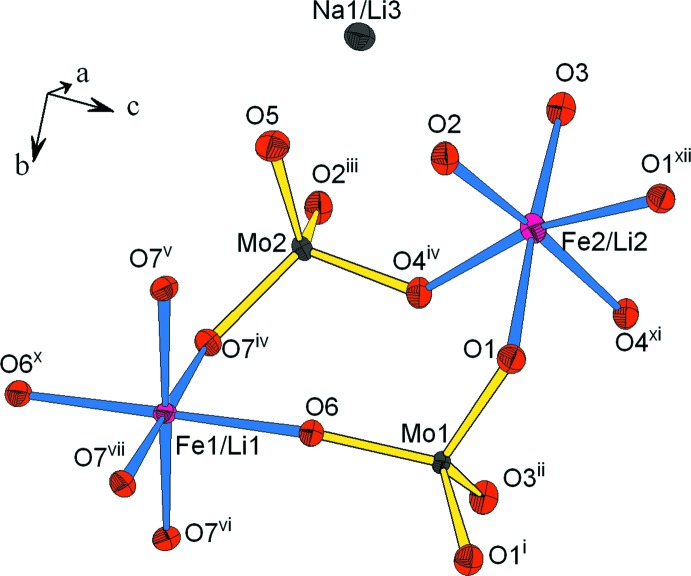
Représentation des polyèdres de coordination de l’unité structurale dans le composé (Na_0,4_,Li_0,6_)(Fe,Li_2_)(MoO_4_)_3_. [Codes de symétrie: (i) *x*, −*y* + 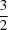, *z*; (ii) *x*, *y*, *z* + 1; (iii) *x* + 1, *y*, *z*; (iv) *x*, *y* + 1, *z*; (v) *x* − 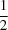, −*y* + 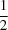, −*z* + 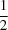; (vi) *x* − 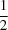, *y*, −*z* + 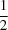; (vii) *x* − 1, *y*, *z*; (x) −*x* + 1, −*y* + 1, −*z* + 1; (xi) −*x* + 1, −*y* + 1, −*z*; (xii) *x* − 1, *y*, *z* − 1.]

**Figure 2:**
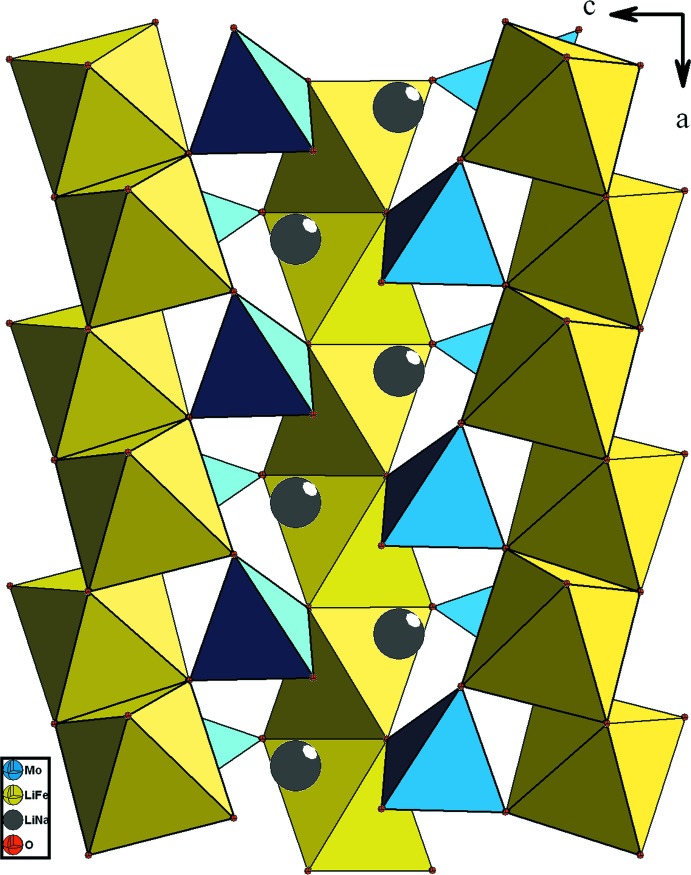
Forme d’un ruban selon *a* dans le composé (Na_0,4_,Li_0,6_)(Fe,Li_2_)(MoO_4_)_3_.

**Figure 3:**
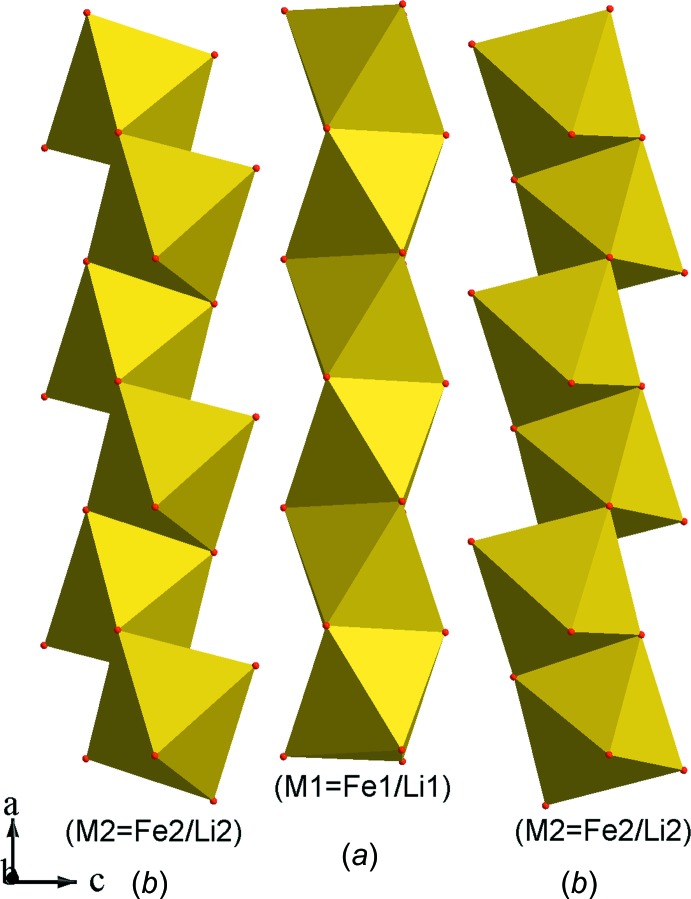
Chaînes d’octa­èdres dans le composé (Na_0,4_,Li_0,6_)(Fe,Li_2_)(MoO_4_)_3_. (*a*): type 1; (*b*): type 2.

L’unité structurale dans la charpente anionique du composé étudié (Fig. 1 ▸) est formée de deux octa­èdres *M*O_6_ (*M* = Fe/Li), disposés en cycle avec deux tétraèdres MoO_4_ liés par mise en commun de sommets oxygène. Ces unités se regroupent par partage d’arêtes entre octa­èdres ainsi que par formation de ponts mixtes de type *M*1—O—Mo (*M*1 = Fe1/Li1) pour conduire à des rubans disposés selon la direction [100] (Fig. 2 ▸). Dans la charpente anionique les octa­èdres se lient de deux façons différentes pour conduire à deux types de chaînes. En effet, les octa­èdres *M*1O_6_ (*M*1 = Fe1/Li1) se regroupent moyennant des faces pour donner naissance à des chaînes de type (*M*1O_3_) (*M*1 = Fe1/Li1) (Fig. 3 ▸ *a*) dans lesquelle la distance métal–métal s’avère très courte de l’ordre 2,57 Å, qui pourrait prédire certaines propriétés magnétiques au matériau obtenu. Par contre les octa­èdres *M*2O_6_ se connectent par mise en commun d’arêtes pour former des chaînes de type *M*2O_4_ (*M*2 = Fe2/Li2) (Fig. 3 ▸ *b*). Dans ces dernières les distances métal–métal sont situées dans l’inter­val (3,050—3,197 Å) similaires à celles rencontrées dans les matériaux CsFe_5_(MoO_4_)_7_ (Namsaraeva *et al.*, 2011 ▸) et K_2_Zn_2_(MoO_4_)_3_ (Gicquel-Mayer & Perez 1975 ▸).

**Figure 4:**
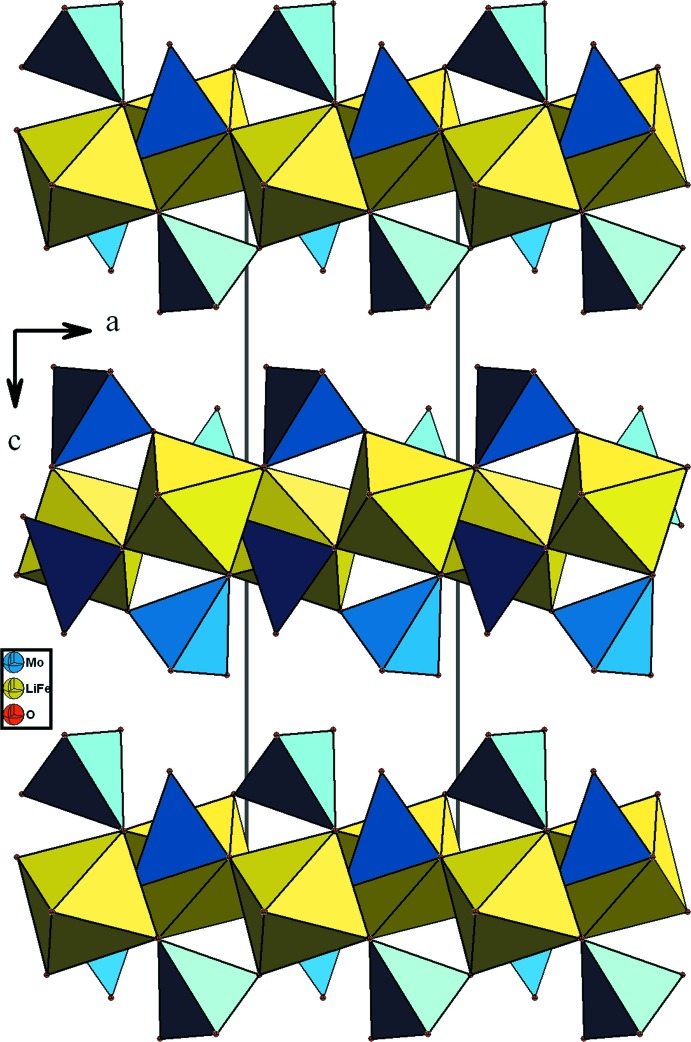
Vue selon *b*, montrant la disposition des couches dans le composé (Na_0,4_,Li_0,6_)(Fe,Li_2_)(MoO_4_)_3_.

**Figure 5:**
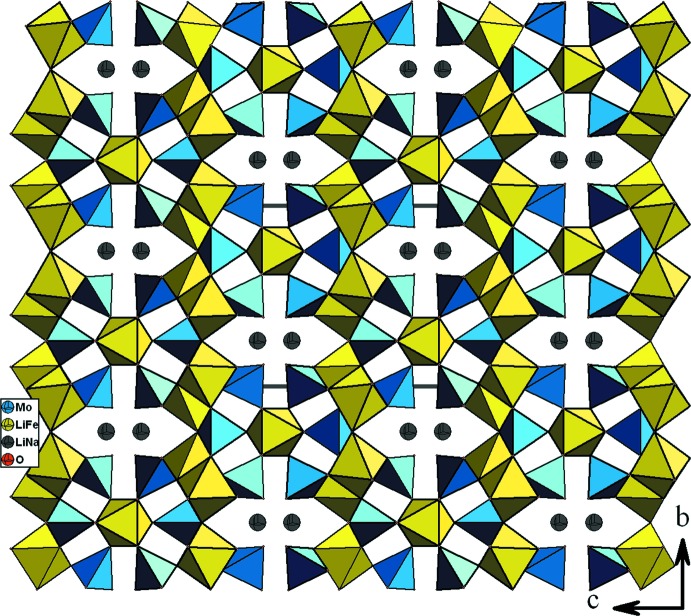
Projection de la structure de (Na_0,4_,Li_0,6_)(Fe,Li_2_)(MoO_4_)_3_, selon *a*, mettant en évidence la disposition des cations alcalins.

Les chaînes s’associent par partage de sommets avec les tétraèdres MoO_4_ pour conduire à des couches disposées parallèlement au plan *ab* (Fig. 4 ▸). La jonction de ces couches est assurée, d’une part par insertion des chaînes de type bronze *M*1O_3_ (*M*1 = Fe1/Li1), et d’autre part par partage de sommet avec les tétraèdres MoO_4_. Il en résulte une charpente tri­dimensionnelle possédant des canaux allongés où résident les cations monovalents (Fig. 5 ▸).

L’examen des distances Mo—O existant dans les tétraèdres MoO_4_ (tableau 1 ▸) sont en bon accord avec celles rencontrées dans la littérature (Klevtsova & Magarill, 1970 ▸; van der Lee *et al.*, 2008 ▸).

Par contre, les facteurs métriques des octa­èdres *M*O_6_ dans la structure s’avèrent des distances moyennes entre celles Fe^3+^—O et Li^+^—O rencontrèes respectivement dans Klevtsova & Magarill (1970 ▸) et van der Lee *et al.* (2008 ▸).

D’autre part, le calcul des valences de liaisons (BVS), utilisant la formule empirique de Brown (Brown & Altermatt, 1985 ▸; Brown, 2002 ▸), conduit aux valeurs des charges des cations suivants: Fe1/Li1 2,73; Fe2/Li2 2,37; Mo1 5,68; Mo2 5,75 et Na1/Li3 1,06.

## Enquête de base de données   

Dans le cadre d’élaboration de nouveaux molybdates de fer, ainsi que l’amélioration des performances électrochimiques de ces batteries, on a voulu substituer le lithium par le sodium d’où la synthèse de notre nouvelle phase de formulation (Na_0,4_,Li_0,6_)(Li_2_,Fe)(MoO_4_)_3_. Une recherche bibliographique des paramètres de maille dans la base de données Findit (ICSD, 2007 ▸) montre que la phase élaborée est isotype à deux structures: Li_3_Fe(MoO_4_)_3_ (Klevtsova & Magarill, 1970 ▸) et Li_3_Ga(MoO_4_)_3_ (van der Lee *et al.*, 2008 ▸). Elle est formée d’une charpente tridimensionnelle construite à partir d’octa­èdres *M*O_6_ (*M* = Fe/Li) et des tétraèdres MoO_4_.

**Figure 6:**
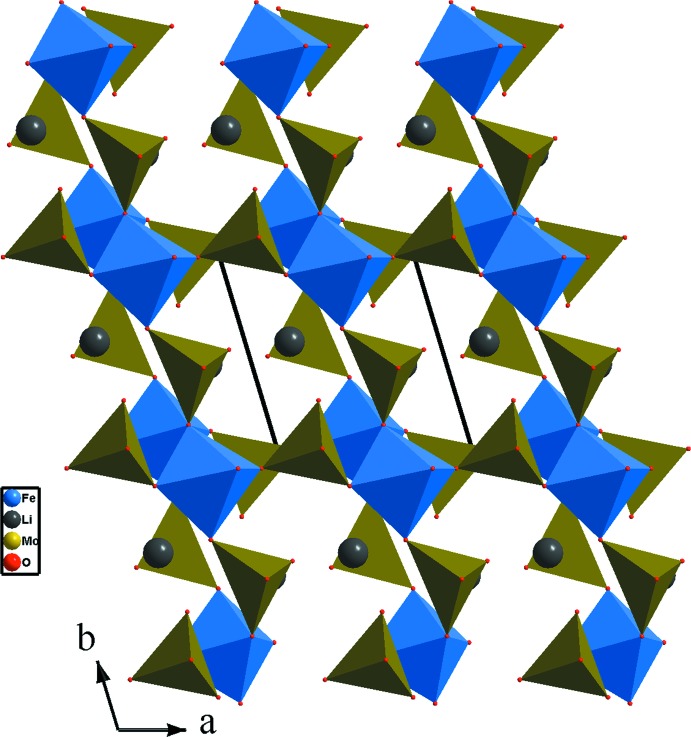
Jonction des polyèdres dans la structure de LiFe(MoO_4_)_2_.

**Figure 7:**
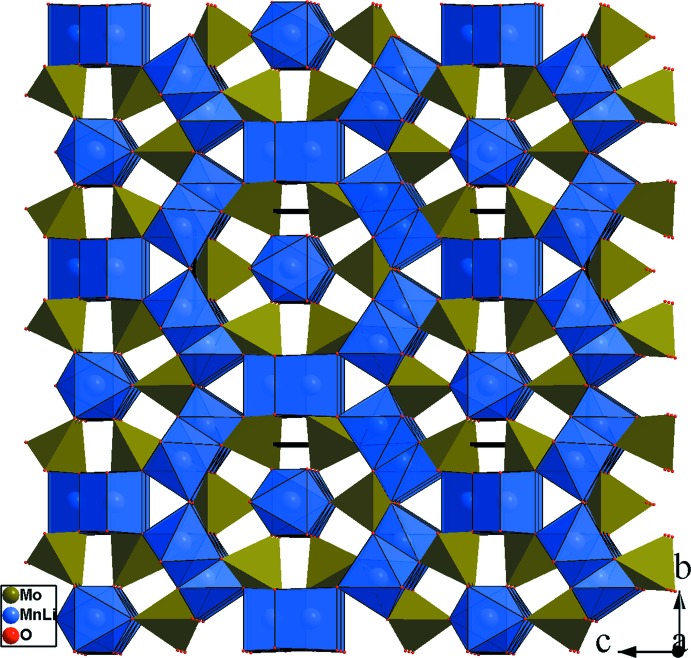
Projection de la structure de Li_1.6_Mn_2.2_Mo_3_O_12_, selon *a*, montrant la disposition des ions Mn/Li dans les octa­èdres et dans les bipyramides.

Une comparaison de la structure du composé étudié Na_0,4_Li_2,6_Fe(MoO_4_)_3_ avec celle de LiFeMo_2_O_8_ (van der Lee *et al.*, 2008 ▸) de système cristallin triclinique (groupe d’espace *P* 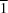) montre une différence nette dans l’entourage des polyèdres et en particulier les types de chaînes dans la charpente anionique. En effet, on remarque que pour le composé au lithium, chaque molybdate MoO_4_ permet de relier par partage de sommets deux octa­èdres FeO_6_ différents appartenant à la même chaîne (Fig. 6 ▸). Par contre dans le composé étudié chaque molybdate partage trois de ses sommets avec trois octa­èdres *M*2O_6_ pour relier deux types de chaînes différentes. Un autre examen bibliographique nous a conduit vers la série des phases isostructurales suivantes: Li_3_GaMo_3_O_12_ (van der Lee *et al.*, 2008 ▸), Li_3_FeMo_3_O_12_ (Klevtsova & Magarill, 1970 ▸), Li_3_Ti_0,75_Mo_3_O_12_ (Smit *et al.*, 2008 ▸), et Li_1,6_Mn_2,2_Mo_3_O_12_ (Solodovnikov *et al.*, 1994 ▸). Ces dernières cristallisent dans le système orthorhombique (groupe d’espace *Pnma*) et appart­iennent à deux familles différentes, notamment les lyonsites et les bronzes. Elles présentent le même type de charpente anionique, sauf pour la dernière phase Li_1,6_Mn_2,2_Mo_3_O_12_ on note bien une légère différence dans l’occupation des sites où les ions (Mn/Li) occupent les sites octa­édriques et aussi les cavités bipyramidales (Fig. 7 ▸).

## Synthèse et cristallisation   

La synthèse de Na_0,4_Li_2,6_Fe(MoO_4_)_3_ a été effectuée par réaction à l’état solide, à partir d’un mélange de carbonate de sodium (FLUKA, 71350), carbonate de lithium (AZIENDA CHIMICA, 104094819), de nitrate de fer (Fluka 44949) et de molybdate d’ammonium (Fluka, 69858) pris dans les rapports molaires telques Na:Li:Fe:Mo égaux à 1:2:1:6, respectivement. Après un broyage poussé dans un mortier en agate, le mélange est placé dans un creuset en porcelaine, puis porté dans un premier temps à une température de 673 K pendant 4 heures, en vue d’éliminer les produits volatils. Un second traitement thermique a été effectué à une température de synthèse proche de la fusion à 1173 K pendant deux semaines pour favoriser la germination des cristaux. Le résidu final est ensuite ramené à un refroidissement lent de 5 K/12 h jusqu’à 1123 K puis rapide (50 K/h) jusqu’à la température ambiante. Des cristaux de couleur verdâtre sont séparés à l’eau chaude.

## Affinement   

Détails de donnés crystallines, collection de donnés et affinement sont résumés dans le tableau 2 ▸. La structure a été résolu par les méthodes directes (*SHELXS97*; Sheldrick, 2008 ▸), partant de la formule NaLi_2_Fe(MoO_4_)_3_ similaire au composé isotype Li_3_Ga(MoO_4_)_3_. Au départ, l’affinement à été mené avec un taux complet des sites. Un examen de la Fourier-différence montre des pics d’intensité (−4,5 Å) proches des atomes de fer (à 0.47 Å from Fe2). L’utilisation des fonctions SUMP et EADP autorisées par le programme *SHELX*, pour les couples d’ions Fe1/Li1, Fe2/Li2 et Na1/Li3 conduit à des ellipsoides bien définis. De plus, les densités d’électrons maximum et minimum restants dans la Fourier-différence sont acceptables et sont situées respectivements à 0.81 Å de O2 et à 0.43 Å de Li2.

## Supplementary Material

Crystal structure: contains datablock(s) I. DOI: 10.1107/S2056989015008737/hb7397sup1.cif

Structure factors: contains datablock(s) I. DOI: 10.1107/S2056989015008737/hb7397Isup2.hkl

CCDC reference: 1063336

Additional supporting information:  crystallographic information; 3D view; checkCIF report

## Figures and Tables

**Table 1:** Longueurs de liaison slectionnes

Na1O2	2,181(4)	Fe2O1^viii^	2,126(3)
Na1O5	2,239(4)	Fe2O3	2,143(3)
Na1O5^i^	2,331(5)	Fe2O1^ix^	2,211(3)
Fe1O7^ii^	2,024(3)	Mo1O1	1,764(3)
Fe1O7^iii^	2,033(3)	Mo1O6	1,786(4)
Fe1O6^iv^	2,094(4)	Mo1O3^x^	1,795(4)
Fe1O6^v^	2,107(4)	Mo2O5	1,735(3)
Fe2O2	2,042(3)	Mo2O2^xi^	1,773(3)
Fe2O4^vi^	2,043(3)	Mo2O4^vii^	1,790(3)
Fe2O4^vii^	2,079(3)	Mo2O7^vii^	1,797(3)

Codes de symtrie: (i) 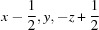; (ii) 
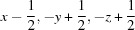
; (iii) 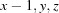; (iv) 
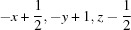
; (v) 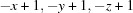; (vi) 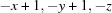; (vii) 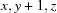; (viii) 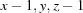; (ix) 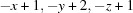; (x) 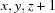; (xi) 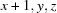.

**Table 2:** Dtails exprimentaux

Donnes crystallines	
Formule chimique	(Na_0,4_Li_0,6_)(FeLi_2_)(MoO_4_)_3_
*M*_r_	562,91
Systme cristallin, groupe d’espace	Orthorhombique, *P* *n* *m* *a*
Temprature (K)	298
*a*, *b*, *c* ()	5,1358(7), 10,5687(9), 17,606(2)
*V* (^3^)	955,61(19)
*Z*	4
Type de rayonnement	Mo *K*
(mm^1^)	5,41
Taille des cristaux (mm)	0,28 0,21 0,14

Collection de donnes	
Diffractomtre	EnrafNonius CAD-4
Correction d’absorption	scan (North *et al.*, 1968 ▸)
*T*_min_, *T*_max_	0,286, 0,488
Nombre de rflexions mesures, indpendantes et observes [*I* > 2(*I*)]	2321, 1091, 1017
*R*_int_	0,023
(sin /)_max_ (^1^)	0,638

Affinement	
*R*[*F* ^2^ > 2(*F* ^2^)], *wR*(*F* ^2^), *S*	0,023, 0,056, 1,16
Nombre de rflexions	1091
Nombre de paramtres	101
_max_, _min_ (e ^3^)	0,52, 0,60

Programmes informatiques: *CAD-4 EXPRESS* (Duisenberg, 1992 ▸; Macek Yordanov, 1992 ▸), *XCAD4* (Harms Wocadlo, 1995 ▸), *SHELXS97* et *SHELXL97* (Sheldrick, 2008 ▸), *DIAMOND* (Brandenburg Putz, 1999 ▸) et *WinGX* (Farrugia, 2012 ▸).

## supplementary crystallographic information

### Crystal data

**Table:**

(Na_0.4_Li_0.6_)(FeLi_2_)(MoO_4_)_3_	*F*(000) = 1041
*M**_r_* = 562.91	*D*_x_ = 3.913 Mg m^−^^3^
Orthorhombic, *P**n**m**a*	Mo *K*α radiation, λ = 0.71073 Å
Hall symbol: -P 2 ac 2n	Cell parameters from 25 reflections
*a* = 5.1358 (7) Å	θ = 11–15°
*b* = 10.5687 (9) Å	µ = 5.41 mm^−^^1^
*c* = 17.606 (2) Å	*T* = 298 K
*V* = 955.61 (19) Å^3^	Prism, green
*Z* = 4	0.28 × 0.21 × 0.14 mm

### Data collection

**Table:**

Enraf–Nonius CAD-4 diffractometer	1017 reflections with *I* > 2σ(*I*)
Radiation source: fine-focus sealed tube	*R*_int_ = 0.023
Graphite monochromator	θ_max_ = 27.0°, θ_min_ = 2.3°
ω/2θ scans	*h* = −6→6
Absorption correction: ψ scan (North *et al.*, 1968)	*k* = −1→13
*T*_min_ = 0.286, *T*_max_ = 0.488	*l* = −1→22
2321 measured reflections	2 standard reflections every 120 min
1091 independent reflections	intensity decay: 1.3%

### Refinement

**Table:**

Refinement on *F*^2^	Primary atom site location: structure-invariant direct methods
Least-squares matrix: full	Secondary atom site location: difference Fourier map
*R*[*F*^2^ > 2σ(*F*^2^)] = 0.023	*w* = 1/[σ^2^(*F*_o_^2^) + (0.0221*P*)^2^ + 2.5918*P*] where *P* = (*F*_o_^2^ + 2*F*_c_^2^)/3
*wR*(*F*^2^) = 0.056	(Δ/σ)_max_ = 0.001
*S* = 1.16	Δρ_max_ = 0.52 e Å^−^^3^
1091 reflections	Δρ_min_ = −0.60 e Å^−^^3^
101 parameters	Extinction correction: *SHELXL*, Fc^*^=kFc[1+0.001xFc^2^λ^3^/sin(2θ)]^-1/4^
0 restraints	Extinction coefficient: 0.0030 (2)

### Special details

**Table:**

Geometry. All e.s.d.'s (except the e.s.d. in the dihedral angle between two l.s. planes) are estimated using the full covariance matrix. The cell e.s.d.'s are taken into account individually in the estimation of e.s.d.'s in distances, angles and torsion angles; correlations between e.s.d.'s in cell parameters are only used when they are defined by crystal symmetry. An approximate (isotropic) treatment of cell e.s.d.'s is used for estimating e.s.d.'s involving l.s. planes.
Refinement. Refinement of *F*^2^ against ALL reflections. The weighted *R*-factor *wR* and goodness of fit *S* are based on *F*^2^, conventional *R*-factors *R* are based on *F*, with *F* set to zero for negative *F*^2^. The threshold expression of *F*^2^ > σ(*F*^2^) is used only for calculating *R*-factors(gt) *etc*. and is not relevant to the choice of reflections for refinement. *R*-factors based on *F*^2^ are statistically about twice as large as those based on *F*, and *R*- factors based on ALL data will be even larger.

### Fractional atomic coordinates and isotropic or equivalent isotropic displacement parameters (Å^2^)

**Table:**

	*x*	*y*	*z*	*U*_iso_*/*U*_eq_	Occ. (<1)
Mo1	0.72100 (8)	0.7500	0.94298 (2)	0.00764 (13)	
Mo2	0.78223 (6)	0.97805 (3)	0.155036 (18)	0.00962 (13)	
Fe1	0.1092 (3)	0.2500	0.24993 (10)	0.0111 (3)	0.40
Li1	0.1092 (3)	0.2500	0.24993 (10)	0.0111 (3)	0.60
Fe2	0.2587 (3)	0.92781 (16)	0.02588 (9)	0.0143 (3)	0.30
Li2	0.2587 (3)	0.92781 (16)	0.02588 (9)	0.0143 (3)	0.70
Na1	0.2569 (8)	0.7500	0.1927 (3)	0.0170 (9)	0.40
Li3	0.2569 (8)	0.7500	0.1927 (3)	0.0170 (9)	0.60
O1	0.9187 (5)	0.8825 (3)	0.96258 (15)	0.0164 (6)	
O2	0.0596 (5)	0.8926 (3)	0.12403 (15)	0.0175 (6)	
O3	0.4481 (8)	0.7500	0.0067 (2)	0.0163 (8)	
O4	0.5823 (6)	0.0138 (3)	0.07454 (15)	0.0152 (6)	
O5	0.5927 (6)	0.8810 (3)	0.21219 (16)	0.0199 (6)	
O6	0.6384 (8)	0.7500	0.8444 (2)	0.0142 (8)	
O7	0.8577 (5)	0.1209 (3)	0.20581 (15)	0.0142 (6)	

### Atomic displacement parameters (Å^2^)

**Table:**

	*U*^11^	*U*^22^	*U*^33^	*U*^12^	*U*^13^	*U*^23^
Mo1	0.0071 (2)	0.0096 (2)	0.0062 (2)	0.000	0.00008 (15)	0.000
Mo2	0.00910 (19)	0.01045 (19)	0.00929 (18)	−0.00100 (12)	−0.00011 (11)	−0.00250 (12)
Fe1	0.0173 (8)	0.0076 (7)	0.0084 (7)	0.000	−0.0018 (7)	0.000
Li1	0.0173 (8)	0.0076 (7)	0.0084 (7)	0.000	−0.0018 (7)	0.000
Fe2	0.0129 (7)	0.0160 (8)	0.0140 (7)	−0.0010 (6)	0.0003 (6)	−0.0022 (7)
Li2	0.0129 (7)	0.0160 (8)	0.0140 (7)	−0.0010 (6)	0.0003 (6)	−0.0022 (7)
Na1	0.017 (2)	0.014 (2)	0.020 (2)	0.000	−0.0016 (16)	0.000
Li3	0.017 (2)	0.014 (2)	0.020 (2)	0.000	−0.0016 (16)	0.000
O1	0.0166 (13)	0.0169 (13)	0.0158 (13)	−0.0032 (12)	−0.0007 (11)	0.0005 (11)
O2	0.0178 (13)	0.0197 (15)	0.0150 (13)	0.0020 (13)	−0.0013 (11)	−0.0020 (11)
O3	0.0116 (18)	0.022 (2)	0.0152 (18)	0.000	0.0010 (15)	0.000
O4	0.0140 (13)	0.0195 (14)	0.0122 (12)	−0.0029 (11)	−0.0001 (11)	−0.0004 (11)
O5	0.0181 (14)	0.0212 (15)	0.0204 (14)	−0.0054 (13)	0.0021 (11)	0.0036 (12)
O6	0.018 (2)	0.0130 (19)	0.0115 (18)	0.000	−0.0017 (15)	0.000
O7	0.0169 (13)	0.0123 (13)	0.0134 (13)	0.0007 (12)	0.0001 (10)	−0.0021 (11)

### Geometric parameters (Å, º)

**Table:**

Na1—O2^i^	2.181 (4)	Fe2—O4^ix^	2.043 (3)
Na1—O2	2.181 (4)	Fe2—O4^x^	2.079 (3)
Na1—O5	2.239 (4)	Fe2—O1^xi^	2.126 (3)
Na1—O5^i^	2.239 (4)	Fe2—O3	2.143 (3)
Na1—O5^ii^	2.331 (5)	Fe2—O1^xii^	2.211 (3)
Na1—O5^iii^	2.331 (5)	Mo1—O1	1.764 (3)
Fe1—O7^iv^	2.024 (3)	Mo1—O1^i^	1.764 (3)
Fe1—O7^ii^	2.024 (3)	Mo1—O6	1.786 (4)
Fe1—O7^v^	2.033 (3)	Mo1—O3^xiii^	1.795 (4)
Fe1—O7^vi^	2.033 (3)	Mo2—O5	1.735 (3)
Fe1—O6^vii^	2.094 (4)	Mo2—O2^xiv^	1.773 (3)
Fe1—O6^viii^	2.107 (4)	Mo2—O4^x^	1.790 (3)
Fe2—O2	2.042 (3)	Mo2—O7^x^	1.797 (3)
			
O1—Mo1—O1^i^	105.12 (19)	O7^vi^—Fe1—O6^vii^	85.29 (13)
O1—Mo1—O6	109.07 (11)	O7^iv^—Fe1—O6^viii^	85.15 (13)
O1^i^—Mo1—O6	109.07 (11)	O7^ii^—Fe1—O6^viii^	85.15 (12)
O1—Mo1—O3^xiii^	109.09 (12)	O7^v^—Fe1—O6^viii^	95.14 (12)
O1^i^—Mo1—O3^xiii^	109.09 (12)	O7^vi^—Fe1—O6^viii^	95.14 (12)
O6—Mo1—O3^xiii^	114.94 (18)	O6^vii^—Fe1—O6^viii^	179.4 (2)
O5—Mo2—O2^xiv^	109.15 (14)	O2—Fe2—O4^ix^	170.95 (15)
O5—Mo2—O4^x^	105.19 (13)	O2—Fe2—O4^x^	97.55 (13)
O2^xiv^—Mo2—O4^x^	108.95 (13)	O4^ix^—Fe2—O4^x^	84.55 (13)
O5—Mo2—O7^x^	109.21 (13)	O2—Fe2—O1^xi^	89.50 (12)
O2^xiv^—Mo2—O7^x^	114.08 (13)	O4^ix^—Fe2—O1^xi^	86.71 (12)
O4^x^—Mo2—O7^x^	109.88 (12)	O4^x^—Fe2—O1^xi^	166.17 (14)
O7^iv^—Fe1—O7^ii^	84.76 (17)	O2—Fe2—O3	101.57 (14)
O7^iv^—Fe1—O7^v^	179.64 (16)	O4^ix^—Fe2—O3	86.97 (14)
O7^ii^—Fe1—O7^v^	95.46 (12)	O4^x^—Fe2—O3	94.90 (14)
O7^iv^—Fe1—O7^vi^	95.46 (12)	O1^xi^—Fe2—O3	95.33 (14)
O7^ii^—Fe1—O7^vi^	179.64 (16)	O2—Fe2—O1^xii^	83.18 (12)
O7^v^—Fe1—O7^vi^	84.32 (17)	O4^ix^—Fe2—O1^xii^	88.31 (13)
O7^iv^—Fe1—O6^vii^	94.41 (13)	O4^x^—Fe2—O1^xii^	83.98 (12)
O7^ii^—Fe1—O6^vii^	94.41 (13)	O1^xi^—Fe2—O1^xii^	85.06 (12)
O7^v^—Fe1—O6^vii^	85.29 (13)	O3—Fe2—O1^xii^	175.23 (15)

Symmetry codes: (i) *x*, −*y*+3/2, *z*; (ii) *x*−1/2, *y*, −*z*+1/2; (iii) *x*−1/2, −*y*+3/2, −*z*+1/2; (iv) *x*−1/2, −*y*+1/2, −*z*+1/2; (v) *x*−1, *y*, *z*; (vi) *x*−1, −*y*+1/2, *z*; (vii) −*x*+1/2, −*y*+1, *z*−1/2; (viii) −*x*+1, −*y*+1, −*z*+1; (ix) −*x*+1, −*y*+1, −*z*; (x) *x*, *y*+1, *z*; (xi) *x*−1, *y*, *z*−1; (xii) −*x*+1, −*y*+2, −*z*+1; (xiii) *x*, *y*, *z*+1; (xiv) *x*+1, *y*, *z*.
